# Effectiveness and safety of tofacitinib in rheumatoid arthritis: a cohort study

**DOI:** 10.1186/s13075-018-1539-6

**Published:** 2018-03-23

**Authors:** Marina Amaral de Ávila Machado, Cristiano Soares de Moura, Steve Ferreira Guerra, Jeffrey R. Curtis, Michal Abrahamowicz, Sasha Bernatsky

**Affiliations:** 1 Division of Clinical Epidemiology, Research Institute of McGill University Health Centre, 5252 de Maisonneuve West, Montreal, QC Canada; 2 Division of Clinical Immunology and Rheumatology, University of Alabama at Birmingham, 619 19th Street South, Birmingham, AL SRC 076 USA; 3 Department of Epidemiology, Biostatistics and Occupational Health, Research Institute of McGill University Health Centre, 5252 de Maisonneuve West, Montreal, QC Canada

**Keywords:** Rheumatoid arthritis, Tofacitinib, Disease-modifying antirheumatic drug, Biologic therapy, Comparative effectiveness research

## Abstract

**Background:**

Tofacitinib is the first oral Janus kinase inhibitor approved for the treatment of rheumatoid arthritis (RA). We compared the effectiveness and safety of tofacitinib, disease-modifying antirheumatic drugs (DMARDs), tumor necrosis factor inhibitors (TNFi), and non-TNF biologics in patients with RA previously treated with methotrexate.

**Methods:**

We used MarketScan® databases (2011–2014) to study methotrexate-exposed patients with RA who were newly prescribed tofacitinib, DMARDs other than methotrexate, and biologics. The date of first prescription was defined as the cohort entry. The therapy was considered effective if all of the following criteria from a claims-based algorithm were achieved at the first year of follow-up: high adherence, no biologic or tofacitinib switch or addition, no DMARD switch or addition, no increase in dose or frequency of index drug, no more than one glucocorticoid joint injection, and no new/increased oral glucocorticoid dose. The safety outcome was serious infections requiring hospitalization. Non-TNF biologics comprised the reference group.

**Results:**

We included 21,832 patients with RA, including 0.8% treated with tofacitinib, 24.7% treated with other DMARDs, 61.2% who had started therapy with TNFi, and 13.3% treated with non-TNF biologics. The rates of therapy effectiveness were 15.4% for tofacitinib, 11.1% for DMARDs, 18.6% for TNFi, and 19.8% for non-TNF biologics. In adjusted analyses, tofacitinib and non-TNF biologics appeared to have similar effectiveness rates, whereas DMARD initiators were less effective than non-TNF biologics. We could not clearly establish if tofacitinib was associated with a higher rate of serious infections.

**Conclusions:**

In patients with RA previously treated with methotrexate, our comparisons of tofacitinib with non-TNF biologics, though not definitive, did not demonstrate differences with respect to hospitalized infections or effectiveness.

**Electronic supplementary material:**

The online version of this article (10.1186/s13075-018-1539-6) contains supplementary material, which is available to authorized users.

## Background

New strategies have been developed to treat rheumatoid arthritis (RA) by inhibiting Janus kinase (JAK) pathways. The first JAK inhibitor approved for the treatment of RA in the United States (November 2012) was tofacitinib. This drug is an oral small molecule and recommended by both the American College of Rheumatology (ACR) and the European League Against Rheumatism (EULAR) as an alternative to biologic drugs when a patient remains with moderate or high disease activity after a first-line disease-modifying antirheumatic drug (DMARD), usually methotrexate, in patients with established RA [1, 2].

Indirect comparisons have suggested that patients with RA in whom DMARD treatment fails experience similar efficacy in response to tumor necrosis factor inhibitors (TNFi), abatacept, tocilizumab, or tofacitinib (monotherapy or in combination with methotrexate). However, caution must be taken when interpreting that result because indirect comparisons are potentially biased [3]. Researchers studying a cohort derived from U.S. administrative databases (2012–2014) reported that patients using tofacitinib, etanercept, adalimumab, and abatacept had similar persistence and adherence at the end of the first year of therapy [4]. In terms of drug safety, it is well known that RA therapies can exacerbate the risk for herpes virus, and evidence from phase III and long-term extension of randomized controlled trials (RCTs) suggests that patients treated with tofacitinib have a higher incidence of herpes virus infection than those treated with placebo or adalimumab [5]. Another systematic review demonstrated that RCTs might not be sensitive enough to compare the risk of adverse events with tofacitinib [6], whereas researchers in one cohort study reported a higher risk for herpes virus infection with tofacitinib than with abatacept, TNFi, and other RA biologics [7]. Thus, the long-term safety of tofacitinib remains mostly uncertain.

More real-world evidence generated during routine clinical practice and obtained outside the context of RCTs is needed [8]. RCTs are typically planned to assess efficacy outcomes and are underpowered for detecting adverse events. Also, follow-up duration in RCTs is short, and long-term extension studies are potentially affected by healthy user bias. Evidence on new drug therapies is required throughout the entire product life cycle, therefore there has been increased interest in comparative effectiveness research on interventions to inform regulatory bodies for health care decision-making and to support optimization of practitioners’ strategies for the management of RA [9].

To provide direct comparative evidence for tofacitinib’s effectiveness and to expand the safety literature as well, we performed a study using real-world population data of tofacitinib, DMARDs, TNFi, and non-TNF biologics for patients with RA. ACR and EULAR recommend tofacitinib after a trial of methotrexate [1, 2]; thus, we studied patients with RA who had been or were exposed to methotrexate.

## Methods

In this retrospective cohort study, we used data from the MarketScan® Commercial Claims and Encounters database and the MarketScan Medicare Supplemental and Coordination of Benefits database (Truven Health Analytics, Ann Arbor, MI, USA) for the period between January 1, 2010, and December 31, 2014. These databases contain claims data for millions of privately insured individuals and their dependents, with many different health plans from large employers, indemnity plans, and health maintenance organizations as well as Medicare-covered patients. These databases include inpatient and outpatient visits, emergency room visits, and outpatient prescription drug information and have been widely used for diverse studies, including RA [10, 11].

We studied adult individuals with RA (at least 18 years old) who were previously treated with methotrexate (oral or subcutaneous) and newly dispensed other DMARDs, biologics, and tofacitinib between January 1, 2011, and December 30, 2014 (*see below* for complete list of drugs). Patients could be currently using methotrexate. The date of the first medication claim, either an outpatient prescription or a procedure claim (in-office drug administration), was defined as the cohort entry. We selected individuals with no use of these medications any time before cohort entry (minimum 12 months), although previous use of DMARDs was allowed for individuals in the biologic and tofacitinib groups. Patients with RA were identified as individuals with at least one inpatient or two outpatient claims (30 to 365 days apart) with an International Classification of Diseases, Ninth Revision, Clinical Modification (ICD-9-CM), code for RA (714.0x or 714.3x). Patients were excluded if they had another diagnosis before cohort entry for which relevant biologics are also approved: ankylosing spondylitis (ICD-9-CM code 720.0x), chronic lymphocytic leukemia (204.1 x), Crohn’s disease (555.xx), juvenile idiopathic arthritis (714.3x), non-Hodgkin’s lymphoma (200.xx, 202.xx), plaque psoriasis (696.1x), psoriatic arthritis (696.0x), or ulcerative colitis (556.xx) [12–14]. We also restricted our analysis to individuals with 12 continuous months of medical and pharmacy coverage prior to cohort entry and for the duration of follow-up.

### Effectiveness analysis

Patients were classified in mutually exclusive groups according to the medication classes dispensed/administered at the cohort entry: (1) DMARDs (sulfasalazine, leflunomide, hydroxychloroquine, and chloroquine), (2) TNFi (adalimumab, etanercept, infliximab, certolizumab, and golimumab) with or without DMARDs, (3) non-TNF biologics (abatacept, rituximab, and tocilizumab) with or without DMARDs, and (4) tofacitinib with or without DMARDs. For this analysis, each patient contributed one therapy episode in one group.

A given therapy was considered effective if the six criteria of a validated claims-based algorithm [14, 15] were achieved throughout 1 year after cohort entry. This algorithm was validated with a gold standard (Disease Activity Score in 28 joints) and presented high sensitivity (72%), specificity (91%), positive predictive value (76%), and negative predictive value (90%) [15]. For this analysis, we considered only patients with at least 1 year of follow-up. The algorithm criteria relate to changes in the initial therapy: (1) high adherence, (2) no biologic or tofacitinib switch or addition, (3) no DMARD switch or addition, (4) no increase in dose or frequency of index drug, (5) no more than one glucocorticoid joint injection, and (6) no new/increased oral glucocorticoid dose. The details of all criteria are presented in Table 1. Adherence to prescribed medication was evaluated using the medication possession ratio (MPR), calculated as the total days’ supply of drug divided by the total follow-up days. Patients with MPRs ≥ 80% were considered highly adherent (i.e., meeting criterion 1).

**Table 1:** Claims-based algorithm for accessing effectiveness after one year of follow-up in patients with rheumatoid arthritis

Criteria	Description of criteria
Criterion 1 High adherence	Adherence to therapy was defined as follows: • For medications with pharmacy claims only: MPR ≥ 80%^a^ • For medications with procedure claims only: Etanercept: ≥ 48 procedures Adalimumab: ≥ 22 procedures Certolizumab: ≥ 22 procedures Golimumab: ≥ 11 procedures Infliximab: ≥ 8 procedures Rituximab: ≥ 4 procedures Abatacept: ≥ 11 procedures Tocilizumab: ≥ 11 procedures
Criterion 2 No prescription or procedure of a new biologic or tofacitinib during follow-up	No prescription or procedure of a new biologic or tofacitinib during follow-up
Criterion 3 No DMARD switch or addition	No prescription of a new DMARD between months 4 and 12 of follow-up
Criterion 4 No increase in dose or frequency of index drug	No increase in dose or frequency of index drug • For medications with pharmacy claims only: ≥ 10% of the daily dose at any time during follow-up compared with the daily dose at cohort entry • For medications with procedure claims only: Adalimumab: > 25 procedures Certolizumab: > 25 procedures Golimumab: > 12 procedures Infliximab: > 9 procedures Rituximab: > 5 procedures Abatacept: > 13 procedures Tocilizumab: > 12 procedures Etanercept: > 55 procedures
Criterion 5 No more than one procedure for glucocorticoid joint injection between months 4 and 12 of follow-up	No more than one procedure for glucocorticoid joint injection between months 4 and 12 of follow-up
Criterion 6 No new/increased oral glucocorticoid dose	No increase in dose of oral glucocorticoid • For patients who received no prescriptions for oral glucocorticoids 6 months prior to cohort entry: > 30 days of cumulative oral glucocorticoid between months 4 and 12 of follow-up • For patients who received prescriptions for oral glucocorticoids 6 months prior to cohort entry: cumulative dose between months 7 and 12 of follow-up ≥ 120% cumulative dose 6 months prior to cohort entry

*MPR* Medication possession ratio, *DMARD* Disease-modifying antirheumatic drug

Adapted from Curtis et al., 2011 [15]

^a^A patient was considered highly adherent if the total days’ supply of drug divided by the total days of follow-up was ≥ 80%

For procedure claims, we used the recommended treatment interval in the U.S. prescribing information to calculate adherence (criterion 1) and increase in biologic dose or frequency (criterion 4) [16–23]. To take into account the differences between prescription and procedure claims regarding the measures of both criteria 1 and 4 (as described above), we excluded patients with a mix of pharmacy and procedure claims for the same index biologic during the follow-up.

In each exposure group, we estimated the separate proportion and the 95% CIs of patients who met individual criteria, as well as the overall proportion of patients for whom the therapy was effective (according to the claims-based algorithm). We estimated adjusted risk ratios with 95% CIs using Poisson regression with robust variance to quantify the relationships between the initiation of treatment with drugs in each exposure group and the effectiveness of the therapy, with non-TNF biologics with or without DMARDs as the reference category, given our particular interest in assessing second-line and subsequent therapies in RA. The models were adjusted for sex, age at entry, and year of cohort entry, as well as for potential confounders measured during 1 year prior to cohort entry: Charlson comorbidity index; hospitalized infection; binary indicators of use of selective cyclooxygenase-2 inhibitors and nonsteroidal anti-inflammatory drugs; oral glucocorticoid use categorized as no use, use of ≤ 7.5 mg/day of prednisone equivalent dose, or ever use of > 7.5 mg/day of prednisone equivalent dose (assessed for each filled prescription); and four indicators of health service use, assessed by the number of (1) emergency department visits, (2) physician visits, (3) rheumatology visits, and (4) hospitalizations.

We performed two sensitivity analyses in which we tested the modified claims-based algorithm for assessing therapy effectiveness while eliminating one of the criteria. First, we excluded criterion 1 (high adherence) to avoid bias estimating adherence of drugs with different administration routes using health claims. Second, we excluded criterion 4 (no increase in dose or frequency of index drug), because patients starting therapy with tofacitinib and some biologics usually do not change the initial dose and/or the frequency.

### Safety analysis

For the safety analysis, current drug exposure was modeled as a time-dependent variable. Accordingly, each subject’s follow-up time was divided into consecutive time intervals with a new interval starting whenever the patient changed, interrupted, and/or started treatment with one of the drug groups of interest. Switching to another drug within the same exposure group was not considered a change of drug exposure. Patients were considered exposed during the days supplied for the prescribed medication or, in the case of an outpatient procedure, the number of days in the recommended treatment interval. We applied a maximum grace period of 90 days, implying that subjects were assumed to remain exposed for the most recently presented drug until 90 days after the end of the prescription or the start of a new drug, whichever came earlier. Within each interval, a patient was classified into one of the same exposure categories as above, plus a no-use category, based on his/her current medication exposure: (1) DMARDs, (2) TNFi with or without DMARDs, (3) non-TNF biologics with or without DMARDs, (4) tofacitinib with or without DMARDs, or (5) nonuse (if not currently in use of any of the drugs in these categories). The outcome was defined as the occurrence of serious infection requiring hospitalization identified from the primary diagnosis recorded in the hospitalization discharge records (ICD-9-CM codes 001-139 or 480–486).

We estimated the rate of serious infections per 100 patient-years for each exposure group. We also estimated the adjusted HRs with 95% CIs, using multivariable time-dependent Cox proportional hazards regression to assess the risk of serious infections associated with the current use of drugs in different categories, represented by time-dependent binary indicators (dummy variables). The non-TNF biologics with or without DMARDs group was the reference category. Adjustments were made for the same covariates described for the effectiveness analysis and additionally for four time-dependent variables indicating current use of methotrexate, current use of glucocorticoid (no use, ≤ 7.5 mg/day, and > 7.5 mg/day), previous use of biologics, and previous use of other DMARDs.

Patients were followed from cohort entry until the earliest date of either (1) the event (i.e., the first occurrence of a serious infection) or (2) censoring at the time of loss of medical or pharmacy coverage, inpatient death, or the end of the study (December 31, 2014). Thus, we considered only one event per patient. In the primary analyses, we allowed patients to continue in the analyses after a drug change placed them in a different exposure category, but in a sensitivity analysis we censored them after they stopped/switched their initial therapy.

We restricted this analysis to only patients covered by Medicare in a sensitivity analysis. The rationale was that patients from commercial health plans enter and leave the cohort according to their drug insurance plan, which can change over time as their employment changes, whereas the Medicare coverage was expected to be more stable. Thus, for commercially insured people, the data we held is only a sampling of their entire health history, and missing information for the remaining periods could have affected the accuracy of the analyses. The Medicare patients, on the other hand, remained in the cohort for the entire follow-up period.

In an additional sensitivity analysis of safety, we repeated our work in a new cohort of patients with RA requiring them to be exposed to biologics and then to have initiated a new biologic agent or tofacitinib. All analyses were conducted using SAS version 9.4 software (SAS Institute, Cary, NC, USA).

## Results

A total of 21,832 patients with RA previously exposed to methotrexate met the inclusion criteria. The therapy at cohort entry included TNFi with or without DMARDs (61.2%), DMARDs alone (24.7%), non-TNF biologics with or without DMARDs (13.3%), and tofacitinib with or without DMARDs (0.8%). Baseline characteristics were similar across groups (Table 2).

**Table 2:** Baseline characteristics of patients with rheumatoid arthritis included in the study

Variable	All patients (*n* = 21,832)	DMARDs (*n* = 5399)	TNFi ± DMARDs (*n* = 13,367)	Non-TNF biologics ± DMARDs (*n* = 2902)	Tofacitinib ± DMARDs (*n* = 164)
Female sex, %	77.0	76.8	76.1	81.4	78.7
Age, years, median (IQR)	56 (48–63)	57 (49–63)	56 (48–63)	58 (50–65)	58 (50–64)
Year of cohort entry, %					
2011	48.5	25.9	52.3	70.5	0
2012	21.6	27.2	20.5	14.1	0.6
2013	14.7	21.9	13.1	8.7	43.9
2014	15.2	25.0	14.1	6.7	55.5
Urban residency, %	82.6	81.1	83.2	82.8	89.4
Oral glucocorticoid use, %					
No use	32.3	29.2	33.4	33.0	31.0
≤ 7.5 mg/day of prednisone equivalent dose	63.0	65.1	62.2	62.2	64.9
> 7.5 mg/day of prednisone equivalent dose	4.7	5.7	4.4	4.8	4.0
Nonsteroidal anti-inflammatory drug use, %	60.7	60.3	62.1	54.9	51.8
Selective COX-2 inhibitor use, %	12.8	10.9	12.9	16.0	13.4
Charlson comorbidity index, mean (SD)	0.61 (0.93)	0.73 (1.04)	0.54 (0.84)	0.73 (1.02)	0.86 (1.14)
Infection-related hospitalization, %	1.6	1.9	1.3	2.6	1.8
Number of emergency department visits, mean (SD)	0.44 (1.20)	0.48 (1.39)	0.42 (1.14)	0.44 (1.05)	0.43 (0.83)
Number of physician visits, mean (SD)	17.99 (13.26)	17.32 (14.07)	17.37 (12.24)	22.21 (15.24)	16.70 (14.24)
Number of rheumatology visits, mean (SD)	4.51 (4.92)	3.39 (3.85)	4.54 (4.71)	6.47 (6.75)	3.88 (3.26)
Number of hospitalizations, mean (SD)	0.16 (0.50)	0.16 (0.54)	0.14 (0.45)	0.23 (0.62)	0.17 (0.62)

*Abbreviations: DMARD* Disease-modifying antirheumatic drug, *TNFi* Tumor necrosis factor inhibitor, *COX-2* Cyclooxygenase-2

Baseline variables were collected at cohort entry (sex, age, year of cohort entry, and place of residence) or 1 year prior to cohort entry (use of drugs, Charlson comorbidity index, hospitalized infection, and indicators of health service use)

### Effectiveness

Overall, therapy effectiveness was rather low, below 20% for each exposure group (first row of Table 3), with the lowest rates being in the DMARDs-alone group (11%). Patients using either TNFi or non-TNF biologics reached better effectiveness rates, followed by tofacitinib. Among the six criteria, high adherence had the lowest rates in all groups and was especially low for DMARDs alone and tofacitinib. For criterion 2 (no biologic or tofacitinib switch or addition), the highest rate observed was among tofacitinib users. For criterion 3 (no DMARD switch or addition), DMARDs had the lowest rate. All patients using tofacitinib met criterion 4 (no dose escalation). Joint injections (criterion 5) occurred less often among patients using DMARDs, and oral glucocorticoid dose increases (criterion 6) were least likely to happen in the TNFi group (Table 3).

**Table 3:** Proportion of patients who achieved therapy effectiveness and individual criteria at 1 year of follow-up (*n* = 16,305)

Effectiveness criteria	DMARDs		TNFi ± DMARDs		Non-TNF biologics ± DMARDs		Tofacitinib ± DMARDs	
	Percent	95% CI	Percent	95% CI	Percent	95% CI	Percent	95% CI
Effective therapy (satisfied all six criteria)	11.1	10.1–12.1	18.6	17.9–19.4	19.8	18.2–21.4	15.4	6.6–24.2
Criterion 1 High adherence	26.6	25.1–28.0	44.0	43.0–44.9	53.3	51.3–55.3	27.7	16.8–38.6
Criterion 2 No biologic or tofacitinib switch or addition	72.7	71.2–74.1	64.3	63.4–65.2	82.1	80.5–83.6	84.6	75.8–93.4
Criterion 3 No DMARD switch or addition	85.3	84.2–86.5	96.1	95.8–96.5	95.5	94.6–96.3	98.5	95.5–100
Criterion 4 No increase in dose or frequency of index drug	92.0	91.1–92.9	94.0	93.5–94.4	88.9	87.6–90.1	100.0^a^	–
Criterion 5 No more than one glucocorticoid joint injection	91.3	90.3–92.2	88.8	88.2–89.4	72.8	71.0–74.6	87.7	79.7–95.7
Criterion 6 No new/increased oral glucocorticoid dose	81.4	80.2–82.7	83.3	82.6–84.1	78.0	76.3–79.7	76.9	66.7–87.2

*DMARD* Disease-modifying antirheumatic drug, *TNFi* Tumor necrosis factor inhibitors

^a^Standard tofacitinib dose is usually not increased

In the multivariable analyses, DMARDs were significantly less likely than non-TNF biologics to be classified as effective (Table 4). For TNFi and the tofacitinib groups, the 95% CIs around the point estimates were fairly wide and precluded definitive conclusions.

**Table 4:** Adjusted risk ratio and 95% CI for medication effectiveness (algorithm result) (*n* = 16,305)

Parameter	Adjusted risk ratio	95% CI
Drug therapy		
Non-TNF biologics ± DMARDs	Reference	–
DMARDs	0.58	0.51–0.66
TNFi ± DMARDs	0.94	0.86–1.03
Tofacitinib ± DMARDs	0.75	0.42–1.34
Sex (female)	0.94	0.87–1.02
Age	1.01	1.00–1.01
Year of cohort entry		
2011	Reference	–
2012	0.87	0.8–0.95
2013	1.00	0.91–1.1
Oral glucocorticoid use 1 year prior to cohort entry		
No use	Reference	–
Use of ≤ 7.5 mg/day of prednisone equivalent dose	0.94	0.88–1.02
Use of > 7.5 mg/day of prednisone equivalent dose	1.07	0.90–1.26
Nonsteroidal anti-inflammatory drug use 1 year prior to cohort entry	0.94	0.88–1.01
Selective COX-2 inhibitors use 1 year prior to cohort entry	0.98	0.88–1.08
Charlson comorbidity index 1 year prior to cohort entry	0.91	0.86–0.95
Infection-related hospitalization 1 year prior to cohort entry	0.84	0.60–1.19
Number of emergency department visits 1 year prior to cohort entry	0.96	0.90–1.01
Number of physician visits 1 year prior to cohort entry	1.00	0.99–1.00
Number of rheumatology visits 1 year prior to cohort entry	1.00	1.00–1.01
Number of hospitalizations 1 year prior to cohort entry	0.97	0.89–1.06

*Abbreviations: DMARD* Disease-modifying antirheumatic drug, *TNFi* Tumor necrosis factor inhibitors, *COX-2* Cyclooxygenase-2

The algorithm without criterion 1 (high adherence) showed that tofacitinib was more likely than non-TNF biologics to reach effectiveness (Additional file 1). When we excluded criterion 4 (no increase in dose or frequency of index drug), most of the multivariate results were similar to the primary analysis, but in addition TNFi were less effective than non-TNF biologics (Additional file 1).

### Safety

Current use of tofacitinib was associated with a rate of serious infections of 3.67 per 100 patient-years, and the 95% CI for this estimate (2.21; 5.75) overlapped with the rate of serious infections for the other drug groups (Table 5). In the multivariate analyses, the hazards of serious infection for DMARDs, TNFi, and tofacitinib were not significant different compared with non-TNF biologic. Current use of methotrexate, previous use of biologics, use of oral glucocorticoid in the year before cohort entry, and current use of oral glucocorticoid were associated with significant increases in the current hazard of infections (Table 6).

**Table 5:** Crude incidence and 95% CIs of serious infection (*n* = 21,832)

Drug therapy	Events	Total person-years	Crude rate (per 100 patient-year)	95% CI
DMARDs	104	5196.02	2.01	1.65–2.42
TNFi ± DMARDs	490	22,736.79	2.16	1.98–2.36
Non-TNF biologic ± DMARDs	173	6936.41	2.49	2.14–2.88
Tofacitinib ± DMARDs	17	474.48	3.67	2.21–5.75

*DMARDs* Disease-modifying antirheumatic drugs, *TNFi* Tumor necrosis factor inhibitors

**Table 6:** Adjusted HRs and 95% CIs for time to serious infection (*n* = 21,832)

Parameter	Adjusted HR	95% CI
Drug therapy		
Non-TNF biologic ± DMARDs	Reference	–
DMARDs	0.80	0.62–1.03
TNFi ± DMARDs	1.14	0.95–1.37
Tofacitinib ± DMARDs	1.54	0.93–2.56
Current use of methotrexate^a^	1.19	1.04–1.37
Previous use of biologics^a^	1.32	1.12–1.57
Previous use of other DMARDs^a^	1.04	0.86–1.27
Sex (female)	1.02	0.87–1.18
Age	1.04	1.03–1.04
Year of cohort entry		
2011	Reference	–
2012	1.10	0.92–1.31
2013	0.85	0.66–1.08
2014	0.97	0.70–1.35
Oral glucocorticoid use one year prior to cohort entry		
No use	Reference	–
Use of ≤ 7.5 mg/day of prednisone equivalent dose	1.23	1.05–1.44
Use of > 7.5 mg/day of prednisone equivalent dose	1.36	1.00–1.84
Current use of oral glucocorticoid^a^		
No use	Reference	–
Use of ≤ 7.5 mg/day of prednisone equivalent dose	1.90	1.64–2.20
Use of > 7.5 mg/day of prednisone equivalent dose	2.83	1.37–5.83
Nonsteroidal anti-inflammatory drugs use 1 year prior to cohort entry	0.93	0.81–1.06
Selective COX-2 inhibitors use 1 year prior to cohort entry	1.13	0.95–1.35
Charlson comorbidity index 1 year prior to cohort entry	1.72	1.49–1.99
Infection related hospitalization 1 year prior to cohort entry	2.19	1.68–2.86
Number of emergency department visits 1 year prior to cohort entry	1.04	1.01–1.06
Number of physician visits 1 year prior to cohort entry	1.01	1.00–1.01
Number of rheumatology visits 1 year prior to cohort entry	1.00	0.99–1.02
Number of hospitalizations 1 year prior to cohort entry	1.33	1.24–1.43

*DMARDs* Disease-modifying antirheumatic drug, *TNFi* Tumor necrosis factor inhibitors

^a^Time-varying covariates

Our findings were similar in the sensitivity analysis, where we censored patients after they discontinued or switched their initial therapy (Additional file 2). The analysis limited to Medicare data reduced the sample to 5200 patients. The incidence of serious infection was higher in the Medicare patients than in the primary analyses, across all exposure groups, but the HRs were generally similar to the primary analysis, despite the wide CIs (Additional file 3). Finally, in an additional sensitivity analysis in a new cohort of patients with RA previously exposed to a biologic agent, the results also indicated that tofacitinib was not significantly different from non-TNF biologics (Additional file 4).

## Discussion

In this retrospective cohort study of patients previously treated with methotrexate, we evaluated the effectiveness and risk of serious infection of tofacitinib compared with DMARDs, TNFi, and non-TNF biologics. Effectiveness at 1 year of follow-up, estimated by a claims-based algorithm, was noted in 15% of tofacitinib users, 11% of DMARD users, and around 19% of TNFi and non-TNF biologic users. The adjusted analysis showed that patients using tofacitinib were as likely as non-TNF biologic users to show effectiveness. The algorithm measures effectiveness on the basis of changes of the initial therapy that can capture both lack of or low effectiveness as well as drug intolerance and side effects. Therefore, it may be expected that our results show lower “success” rates than RCTs. In addition of course, our “real-world” data reflect a different population from the relatively narrow and homogeneous range of subjects enrolled in RCTs, which also will result in different “success” rates as compared with RCTs.

Adherence was an important component in our effectiveness algorithms: less than half of patients in the TNFi and non-TNF biologic groups and only one-third in the tofacitinib and DMARD groups continued their initial therapy. This criterion does not distinguish between actual noncompliance with the treatment and intolerance or adverse events. Switching/addition was another important factor contributing to overall algorithm failure, particularly in the TNFi group. Note that patients using tofacitinib did not increase their initial dose, given that there is only one Food and Drug Administration-approved dose. The adjusted comparisons of the final algorithm result suggested greater effectiveness for TNFi or non-TNF biologics than for DMARDs, and we were unable to establish differences for tofacitinib and DMARDs. In effect, tofacitinib appeared as effective as biologics on the basis of most criteria. Researchers in another cohort study using pharmacy claims reported a similar proportion of persistence and adherence (measured by proportion of days covered) at the first year of therapy among patients using tofacitinib, etanercept, adalimumab, and abatacept. A greater proportion of patients using etanercept and adalimumab switched to tofacitinib or another biologic. Finally, dose increase happened in 5% of patients using tofacitinib [4].

Our study shows that the unadjusted incidence of hospitalizations for infection was 3.6 per 100 patient-years for tofacitinib, compared with 2.2 and 2.5 per 100 patient-years in individuals using TNFi and non-TNF biologics, respectively. Higher rates were observed in each of these groups in the cohort containing only patients covered by Medicare (aged > 65 years). Recent cohort studies showed different point estimates for DMARDs and biologic agents [24–26], which may be explained by the difference in the definition of serious infections, by differences in patients’ profiles regarding other risk factors for infections, or perhaps by chance alone. Our adjusted comparisons did not demonstrate that tofacitinib users had an increased risk of serious (hospitalized) infections compared with those treated with non-TNF biologics. One recent study using Medicare and MarketScan databases showed that patients using tofacitinib had a higher risk for herpes virus infection than those using biologic drugs [7]. Although the aim of our study was not to directly compare infection risk of tofacitinib patients with that of those treated with anti-TNF drugs only, we performed this comparison and did not find a significant difference. Factors that were associated with higher risk of infection in our study were current use of methotrexate, use of oral glucocorticoid before cohort entry, and previous use of biologics.

In our sample, after a first treatment with methotrexate, tofacitinib was not commonly prescribed. Although the ACR and EULAR guidelines recommend the use of tofacitinib after failure of therapy with DMARDs in patients with established RA, others argue that the JAK inhibitors (owing to the uncertainty about long-term safety and cost-effectiveness) should be used only after insufficient response to initial biologic treatment [27]. The dearth of evidence from observational studies to date was in part the motivation for our work in this analysis to inform the comparative effectiveness and safety of tofacitinib. Our additional analysis of tofacitinib after failure of a first biologic therapy for patients with RA showed similar safety in terms of serious (hospitalized) infection.

Our study has potential limitations. First, we obtained a small sample of tofacitinib users, which was expected for a novel therapy. The patients were not clinically confirmed, but we did use a validated RA definition; our patients were all previously exposed to the cornerstone RA therapy (methotrexate); and we excluded patients with other indications for various RA drug exposures (e.g., cancer, other rheumatic diseases). Still, although MarketScan databases collect data on a large population, it lacks data on important clinical variables and reasons for changing the initial therapy. As a result, we indirectly measured therapy effectiveness using an algorithm based on registers of drug prescriptions or drug administration. This algorithm is composed of criteria related to actionable interventions used by physicians. Although the algorithm has previously been validated for anti-TNF and DMARD therapy in RA, it seemed reasonable to apply the same set of criteria to measure the response to non-TNF biologics and tofacitinib as we did in our study. In addition, herpes zoster (which usually does not require hospitalization) is potentially an important infectious outcome but not one we focused on in this analysis.

All observational studies are at risk of “channeling”; that is, persons are not allocated drugs randomly, and the characteristics of certain people who tend to receive certain drugs may become confounders (e.g., RA disease duration and prior biologic use, which have been shown to be confounders of infection risk [28]) if those are also associated with the outcome of interest. So, for example, it may be that people assigned to tofacitinib were more or less likely (because of their past response to drugs in terms of effectiveness or infection) to have the outcome of interest. We aimed to overcome this potential issue by studying only those patients with RA with previous methotrexate exposure, and we also controlled for proxies of disease severity (e.g., past drugs and rheumatology visits).

We note that in the dataset that we used, patients’ follow-up could have happened because of a loss or change in their health insurance. Medicare completely captures patients’ claims, and the analysis restricted to those patients showed similar risk for infections, despite the lower precision.

As a final point that should be taken into account when interpreting and comparing our results, in our analyses, patients using biologics or tofacitinib could also be taking one or more DMARDs, including MTX. However, most of these combinations would be with drugs such as hydroxychloroquine, which itself tends not to increase infection risk.

## Conclusions

In summary, in this report of a large population-based cohort study, we present novel data on real-world use of tofacitinib in patients with RA previously treated with methotrexate. Our study estimated similar (relatively low) effectiveness rates at 1 year of follow-up for patients starting tofacitinib after therapy with methotrexate, as opposed to initiators of non-TNF biologics. Our comparisons of tofacitinib versus non-TNF biologics, though not definitive, did not clearly demonstrate differences with respect to hospitalized infections.

## Additional files

Additional file 1: Proportion and adjusted risk ratio of patients who achieved therapy effectiveness based on the modified algorithm (*n* = 16,305). (DOCX 14 kb)

Additional file 2: Adjusted HR for time to serious infection in patients censored after they stopped/switched their initial therapy (*n* = 21,832). (DOCX 14 kb)

Additional file 3: Adjusted HR for time to serious infection in patients covered by Medicare (*n* = 5200). (DOCX 13 kb)

Additional file 4: Crude incidence of serious infection in patients with RA previously exposed to a biologic agent (*n* = 14,875). Adjusted HR for time to serious infection in patients with RA previously exposed to a biologic agent (*n* = 14,875). (DOCX 15 kb)

## Abbreviations

ACR: American College of Rheumatology
COX-2: Cyclooxygenase-2
DMARD: Disease-modifying antirheumatic drug
EULAR: European League Against Rheumatism
ICD-9-CM: International Classification of Diseases, Ninth Revision, Clinical Modification
JAK: Janus kinase
MPR: Medication possession ratio
RA: Rheumatoid arthritis
RCT: Randomized controlled trial
TNFi: Tumor necrosis factor inhibitor(s)
