# Supplementary material for: Effectiveness and safety of tofacitinib in rheumatoid arthritis: a cohort study
Source: Arthritis Res Ther. 2018 Mar 23;20:60. doi: 10.1186/s13075-018-1539-6 (PMC5865387; doi:10.1186/s13075-018-1539-6)
Supplement: Supplementary file 1 — Proportion and adjusted risk ratio of patients who achieved therapy effectiveness based on the modified algorithm (n = 16,305). (DOCX 14 kb) [file 13075_2018_1539_MOESM1_ESM.docx]

Additional file 1

Proportion and adjusted risk ratio of patients who achieved therapy effectiveness based on the modified algorithm (N=16,305).

| Drug therapy | Effective therapy | | Poisson regression | |
| --- | --- | --- | --- | --- |
|  | % | 95% Confidence Interval | Adjusted risk ratio | 95% Confidence Interval |
| Algorithm with 5 criteria excluding Criterion 1 - high adherence | | | | |
| Non-TNF biologic +/- DMARDs | 40.8 | 38.8; 42.8 | reference | - |
| DMARDs | 46.5 | 44.9; 48.1 | 1.17 | 1.10; 1.24 |
| TNFi +/- DMARDs | 46.7 | 45.7; 47.6 | 1.15 | 1.09; 1.21 |
| Tofacitinib +/- DMARDs | 56.9 | 44.9; 69.0 | 1.49 | 1.19; 1.86 |
| Algorithm with 5 criteria excluding Criterion 4 - no increase in dose or frequency of index drug | | | | |
| Non-TNF biologic +/- DMARDs | 24.9 | 23.2; 26.7 | reference | - |
| DMARDs | 13.0 | 11.9; 14.1 | 0.55 | 0.49; 0.62 |
| TNFi +/- DMARDs | 20.3 | 19.5; 21.0 | 0.82 | 0.76; 0.89 |
| Tofacitinib +/- DMARDs | 15.4 | 6.6; 24.2 | 0.61 | 0.34; 1.09 |

The original algorithm is composed by six criteria: Criterion 1 - High adherence; Criterion 2 - No biologic or tofacitinib switch or addition; Criterion 3 - No DMARD switch or addition; Criterion 4 - No increase in dose or frequency of index drug; Criterion 5 - No more than one glucocorticoid joint injection; Criterion 6 - No new/increase oral glucocorticoid dose.

DMARDs: disease-modifying antirheumatic drug; TNFi: tumor necrosis factor inhibitors.

Risk ratios adjusted for baseline sex, age, year of cohort entry, Charlson comorbity index, hospitalized infection, use of selective cox-2 inhibitors, nonsteroidal anti-inflammatory drugs, and oral glucocorticoid, and number of emergency department visits, physician visits, rheumatology visits, and hospitalizations.

DMARD: disease-modifying antirheumatic drug; TNFi: tumor necrosis factor inhibitors.
