# Supplementary material for: Effectiveness and safety of tofacitinib in rheumatoid arthritis: a cohort study
Source: Arthritis Res Ther. 2018 Mar 23;20:60. doi: 10.1186/s13075-018-1539-6 (PMC5865387; doi:10.1186/s13075-018-1539-6)
Supplement: Supplementary file 2 — Adjusted HR for time to serious infection in patients censored after they stopped/switched their initial therapy (n = 21,832). (DOCX 14 kb) [file 13075_2018_1539_MOESM2_ESM.docx]

Additional file 2

Adjusted hazard ratio for time to serious infection in patients censored after they stopped/switched their initial therapy (N= 21,832).

| Drug therapy | Events | Total person-years | Crude rate (per 100 patient-years) | 95% Confidence Interval | Adjusted Hazard Ratio | 95% Confidence Interval |
| --- | --- | --- | --- | --- | --- | --- |
| Non-TNF biologic +/- DMARDs | 104 | 3998.40 | 2.60 | 2.14; 3.14 | reference | - |
| DMARDs | 73 | 3542.07 | 2.06 | 1.63; 2.58 | 0.69 | 0.50; 0.96 |
| TNFi +/- DMARDs | 385 | 17806.49 | 2.16 | 1.95; 2.39 | 1.12 | 0.90; 1.40 |
| Tofacitinib +/- DMARDs | 3 | 89.87 | 3.34 | 0.85; 9.09 | 1.20 | 0.37; 3.86 |

DMARDs: disease-modifying antirheumatic drug; TNFi: tumor necrosis factor inhibitors.

Hazard ratios adjusted for baseline sex, age, year of cohort entry, Charlson comorbity index, hospitalized infection, use of selective cox-2 inhibitors, nonsteroidal anti-inflammatory drugs, and oral glucocorticoid, and number of emergency department visits, physician visits, rheumatology visits, and hospitalizations. Additionally, Hazard ratios were adjusted for time-varying indicators of current use of methotrexate, current use of glucocorticoid, and previous use of other DMARDs.
