# Supplementary material for: Effectiveness and safety of tofacitinib in rheumatoid arthritis: a cohort study
Source: Arthritis Res Ther. 2018 Mar 23;20:60. doi: 10.1186/s13075-018-1539-6 (PMC5865387; doi:10.1186/s13075-018-1539-6)
Supplement: Supplementary file 3 — Adjusted HR for time to serious infection in patients covered by Medicare (n = 5200). (DOCX 13 kb) [file 13075_2018_1539_MOESM3_ESM.docx]

Additional file 3

Adjusted hazard ratio for time to serious infection in patients covered by Medicare (N= 5,200).

| Drug therapy | Events | Total person-years | Crude rate (per 100 patient-years) | 95% Confidence Interval | Adjusted Hazard Ratio | 95% Confidence Interval |
| --- | --- | --- | --- | --- | --- | --- |
| Non-TNF biologic +/- DMARDs | 87 | 2187.32 | 3.34 | 2.63; 4.17 | reference | - |
| DMARDs | 61 | 1623.19 | 3.79 | 2.92; 4.83 | 0.92 | 0.65; 1.30 |
| TNFi +/- DMARDs | 221 | 5833.58 | 3.78 | 3.31; 4.31 | 1.14 | 0.88; 1.48 |
| Tofacitinib +/- DMARDs | 7 | 121.93 | 3.98 | 3.21; 4.89 | 1.51 | 0.69; 3.31 |

DMARDs: disease-modifying antirheumatic drug; TNFi: tumor necrosis factor inhibitors.

Hazard ratios adjusted for baseline sex, age, year of cohort entry, Charlson comorbity index, hospitalized infection, use of selective cox-2 inhibitors, nonsteroidal anti-inflammatory drugs, and oral glucocorticoid, and number of emergency department visits, physician visits, rheumatology visits, and hospitalizations. Additionally, Hazard ratios were adjusted for time-varying indicators of current use of methotrexate, current use of glucocorticoid, previous use of biologics, and previous use of other DMARDs.
