# Supplementary material for: Effectiveness and safety of tofacitinib in rheumatoid arthritis: a cohort study
Source: Arthritis Res Ther. 2018 Mar 23;20:60. doi: 10.1186/s13075-018-1539-6 (PMC5865387; doi:10.1186/s13075-018-1539-6)
Supplement: Supplementary file 4 — Crude incidence of serious infection in patients with RA previously exposed to a biologic agent (n = 14,875). Adjusted HR for time to serious infection in patients with RA previously exposed to a biologic agent (n = 14,875). (DOCX 15 kb) [file 13075_2018_1539_MOESM4_ESM.docx]

Additional file 4

Crude incidence of serious infection in RA patients previously exposed to a biologic agent (N= 14,875).

| Drug therapy | Events | Total person-years | Crude rate (per 100 patient-year) | 95% Confidence Interval |
| --- | --- | --- | --- | --- |
| TNFi +/- DMARDs | 259 | 2714.05 | 2.64 | 2.34; 2.98 |
| Non-TNF biologic +/- DMARDs | 208 | 9819.32 | 2.71 | 2.37; 3.11 |
| Tofacitinib +/- DMARDs | 29 | 7670.07 | 3.18 | 2.21; 4.58 |

DMARDs: disease-modifying antirheumatic drug; TNFi: tumor necrosis factor inhibitors.

Adjusted hazard ratio for time to serious infection in RA patients previously exposed to a biologic agent (N= 14,875).

| Parameter | Adjusted Hazard Ratio | 95% Confidence Interval | |
| --- | --- | --- | --- |
| Drug therapy |  |  |  |
| Non-TNF biologic +/- DMARDs | reference | - |  |
| TNFi +/- DMARDs | 1.18 | 0.98; 1.41 |  |
| Tofacitinib +/- DMARDs | 1.32 | 0.89; 1.97 |  |
| Current use of DMARD * | 0.81 | 0.67; 0.97 |  |
| Previous use of biologics* |  |  |  |
| One biologic agent | reference | - |  |
| Two biologic agents | 1.16 | 0.93; 1.45 |  |
| Two or more biologic agents | 1.21 | 0.89; 1.64 |  |
| DMARD use one year prior to cohort entry | 0.94 | 0.77; 1.14 |  |
| Sex (female) | 1.08 | 0.89; 1.32 |  |
| Age | 1.03 | 1.02; 1.03 |  |
| Year of cohort entry |  |  |  |
| 2011 | reference | - |  |
| 2012 | 0.98 | 0.80; 1.19 |  |
| 2013 | 0.88 | 0.70; 1.12 |  |
| 2014 | 0.69 | 0.48; 1.00 |  |
| Oral glucocorticoid use one year prior to cohort entry |  |  |  |
| No use | reference | - |  |
| Use of ≤7.5 mg/day | 1.28 | 1.03; 1.58 |  |
| Use of >7.5 mg/day | 1.82 | 1.28; 2.60 |  |
| Current use of oral glucocorticoid* |  |  |  |
| No use | reference | - |  |
| Use of ≤7.5 mg/day | 1.54 | 1.29; 1.85 |  |
| Use of >7.5 mg/day | 2.34 | 1.08; 5.08 |  |
| Nonsteroidal anti-inflammatory drugs use one year prior to cohort entry | 0.96 | 0.81; 1.13 |  |
| Selective cox-2 inhibitors use one year prior to cohort entry | 0.95 | 0.73; 1.24 |  |
| Charlson comorbity index one year prior to cohort entry | 1.63 | 1.37; 1.95 |  |
| Infection related hospitalization one year prior to cohort entry | 1.85 | 1.32; 2.60 |  |
| Number of emergency department visits one year prior to cohort entry | 1.09 | 1.05; 1.14 |  |
| Number of physician visits one year prior to cohort entry | 1.01 | 1.01; 1.02 |  |
| Number of rheumatology visits one year prior to cohort entry | 1.02 | 1.00; 1.04 |  |
| Number of hospitalizations one year prior to cohort entry | 1.18 | 1.04; 1.34 |  |

DMARDs: disease-modifying antirheumatic drug; TNFi: tumor necrosis factor inhibitors.

*Time-varying covariates.
